# Barriers Facing Primary Health Care Physicians When Dealing with Emergency Cases in Jeddah, Saudi Arabia

**DOI:** 10.5539/gjhs.v8n8p192

**Published:** 2015-12-17

**Authors:** Majed A. Aloufi, Marwan A. Bakarman

**Affiliations:** 1 General Directorate of Health, Ministery of Health, Jeddah, Saudi Arabia; 2 Rabigh Faculty of Medicine, King Abdulaziz University, Jeddah, Saudi Arabia

**Keywords:** emergency care, primary care physician, family medicine

## Abstract

**Objectives:**

To estimate the prevalence of emergency cases reporting to Primary Health Care centers (PHC), Jeddah, Saudi Arabia and to explore the barriers facing PHC physicians when dealing with such emergency cases.

**Methods:**

A cross-sectional analytic study, where all physicians working in the PHC of the Ministry of Health (MOH) in Jeddah; were invited to participate (n=247). The study period was from July 2013 till December 2013. Data were collected through two sources. 1- A self-administered questionnaire used to determine the physicians’ perceived competence when dealing with emergency cases. 2- A structured observation sheet used to evaluate availability of equipment, drugs, ambulances and other supporting facilities required to deal with emergency cases.

**Results:**

The response rate was 83.4%. The physicians’ age ranged between 25 and 60 years with a mean ±SD of 34.4±7.5 years. Majority of them (83.5%) did not attend ATLS courses at all whereas 60.7% never attended ACLS courses. The majority (97.1%) had however attended BLS courses. Physicians in the age group 36-45 years, non-Saudi, those who had SBFM, those who reported experience in working in emergency departments and physicians who reported more working years in PHCCs (>5 years) had a significant higher score of perceived level of competence in performing emergency skill scale than others (P<0.05). The prevalence of emergency cases attending PHC in Jeddah (2013) was 5.2%.

**Conclusion:**

Emergency services at PHC in Jeddah are functioning reasonably well, but require fine tuning of services and an upgrade in their quality.

## 1. Introduction

An emergency is “a sudden incident that necessitates urgent and appropriate management to treat its results and avoid its sequelae”. It becomes a health emergency if it results in an unexpected risk to the health of people or the physical environment in which they live (Mahfouz et al., 2007).

Physicians working in the primary health care (PHC) setting may come across at least one emergency case per year. Seizures, asthma, shock and cardiac arrest are among the most common medical emergencies in PHC centers. Usually PHC centers are not fully prepared for these medical emergencies. Therefore, all PHC centers should have a written emergency protocol which guides them through the unexpected emergency case. PHC centers can effectively manage emergency cases by having the correct equipment, education and protocols (Toback, 2007; Shenoi et al., 2013)

Some may feel that physicians gain experience in managing acute emergencies during the secondary care portion of their training in hospitals, but several reasons exist why specific training for the primary care setting is essential. With the expansion of training in general physician for 12 months at the expense of secondary care training; physicians exposed to fewer opportunities to experience acute emergencies due to the lower prevalence in primary care as compaired to secondary care (Britton, 2010).

The context in which most physicians obtain and practice the skills for providing care for acute emergencies, e.g. Advanced Trauma Life Support (ATLS), Paediatric Advanced Life Support (PALS) etc., is in the heart of the hospital network; a network where a team of similarly trained and practiced professionals are present with resources, e.g. defibrillators, emergency medications etc., close to hand. Moreover, the teams get to practice with some regularity these skills on real cases (Britton, 2010).

In contrast to the above, in primary care, although the materials are usually available and maintained in case a situation should arise, these skills are rarely used. Moreover, the supportive practitioners (nurses, fellow clinicians and receptionists) although receiving yearly training; may wait years before they get to practice their skills on a real case. Indeed, the physicians being transferred from the relatively standardized and familiar context of the hospital or outpatient department to a new and much more variable primary care setting, therefore they will be disoriented and relatively isolated if confronted with a patient having a life threatening event. Finally, many physicians will never have taken the responsibility for directing the care or acting in lead clinician role during the emergency (Britton, 2010).

Data regarding emergency services at PHC level in Jeddah are scarce. Relevant data from health service providers, especially physicians, are very important to health care policy-makers for effective and optimal management of the current services.

The researcher adopted the following definition for emergency; it will include not only life-threatening situation but also will include emergency that will be managed totally or partially at PHC centers. Our objectives in this study were to estimate the prevalence of emergency cases reporting to PHC centres in Jeddah, Saudi Arabia and to explore the barriers facing PHC physicians when dealing with such emergency cases.

## 2. Methodology

### 2.1 Research Desigin and Subjects

This is a cross-sectional study where all the primary health care physicians working in the PHC centers of the Ministry Of Health (MOH) in Jeddah, Saudi Arabia during the study period. Forty PHC centres in Jeddah city where distributed in four geographical sectors. There are 218 general physicians and 67 family physicians working in those PHC centers. Total number of patients visiting PHC centers in 2013 was 1,340,553 patients.

### 2.2 Sampling

All the primary health care physicians who work in PHC centres, MOH in Jeddah city, at the period of the study constitute the target population. There total number was 285 physicains.

Thirty eight physicians were out of the work either internal or external. Thus, the total number of physicians invited to participate in the study was 247.

### 2.3 Reseacrch Insturment

The invited physicans were participated by completing a self-administered questionnaire structured by the researchers and validated by three consultants. The questionnaire contain the socio-demographic data, questions to identify level of their training, previous experience and emergency courses, questions to determine their perceived competence when dealing with emergency cases, frequency of emergency cases in the last 12 months and their satisfaction with emergency services provided at their PHC centers.

A structured observation sheet was also used to evaluate availability of equipments, drugs, ambulance and other supporting facilities which are needed to deal with emergency cases. This sheet was structured using the Saudi Ministry of Health Quality assurance manual, (Al-Mazrou & Salem, 1993) the Saudi essential drug list at primary health care level (Al-Mazrou S, 2012) and the Saudi Ministry of Health Primary Health Care manual. (Al-Mazrou et al, 2003).

### 2.4 Data Analysis

Data entry and statistical analyses were done using statistical software package the SPSS version 20. The study was from July 2013 till December 2013.

## 3. Results

Out of 247 PHC physicians recruited for the study, 206 responded by returning back completed questionnaires, a total response rate of 83.4%.

Their age ranged between 25 and 60 years with a mean ±SD of 34.4±7.5 years. Almost two-thirds of them (64.5%) aged between 25 and 35 years. Female physicians represent 59.2%. Most were Saudi (71.8%) and more than two-thirds (69.9%) had obtained MBBS, while only 23 (11.2%) physicians having the fellowship in Family Medicine.

The vast majority of PHC physicians (97.1%) had attended basic life support (BLS) courses, but (83.5%) had not attended ATLS courses, with 60.7% never attending advanced cardiac life support (ACLS) courses. Out of the physicians who attended such courses, 46.1% attended BLS during the last year, whereas 6.8% and 5.3% of them attended ACLS and ATLS last year, respectively.

Most of PHC physicians (72.3%) had previous experience of working in the emergency departments of hospitals.

Throughout the year 2013; there were 1,340,553 patients of varying ages visited the primary health care centers in Jeddah. Of these, 70,284 of these were labelled as emergency cases. Thus the prevalence of emergency cases attending PHC centers in Jeddah in 2013 was 5.2%.

Most of the PHC physicians (70.4%) have seen 3 or more cases of acute asthma in the last 12 months, whereas only 5.8%, 7.8% and 8.7% of the physicians have seen three or more cases of anaphylaxis, myocardial infarction and cardiac arrest in the last 12 months, respectively. More than a third (39.3%) have seen three or more cases of renal colic and almost two thirds (63.1%) did not see any case of myocardial infarction, convulsion (62.1%), anaphylaxis (68.9%) or acute vaginal bleeding (65%). Most (80.6%) did not see any case of cardiac arrest.

As shown in table 1, more than half of PHC physicians (50.5%) will perform neublization and oxygen therapy in all cases, with 44.2% performing simple suturing in all cases. Just over a third of the physicians will attempt cardiac compression (38.8%), bag & mask resuscitation (37.9%) and using intravenous (IV) fluid & medications (36.4%) in all cases. Only 9.7% and 16% of them will attempt intubation or defibrillation respectively, in all cases.

**Table 1:** Perceived level of competence in performing emergency skills among primary health care physicians, MOH, Jeddah

Factors associated with perceived level of competence	Would not know where to start N (%)	Will do if no one else is available N (%)	Will attempt in most cases N (%)	Will attempt in all cases N (%)
Cardiac compression	9 (4.4)	44 (21.4)	73 (35.4)	80 (38.8)
Mouth to mouth resuscitation	21 (10.2)	74 (35.9)	53 (25.7)	58 (28.2)
Bag & mask resuscitation	13 (6.3)	43 (20.9)	72 (35.0)	78 (37.9)
Inserting IV cannula	24 (11.7)	85 (41.3)	50 (24.3)	47 (22.8)
Intubation	71 (34.5)	77 (37.4)	38 (18.4)	20 (9.7)
Defibrillation	53 (25.7)	76 (36.9)	44 (21.4)	33 (16.0)
Reading ECG*	28 (13.6)	49 (23.8)	62 (30.1)	67 (32.5)
Nebulisation & oxygen therapy	13 (6.3)	33 (16.0)	56 (27.2)	104 (50.5)
Simple suturing	25 (12.1)	43 (20.9)	47 (22.8)	94 (44.2)
NGT insertion #	31 (15.0)	67 (32.5)	50 (24.3)	58 (28.2)
Urinary catheter insertion	27 (13.1)	74 (35.9)	42 (20.4)	63 (30.6)
Administering IV fluid & medications**	18 (8.7)	58 (28.2)	55 (26.7)	75 (36.4)

# NGT: Nasogastric tube;

* ECG: Electrocardiogram;

** IV: Intravenous.

Table 2 summarizes the factors associated with the perceived level of competence in performing emergency skills scale. Physicians in the age group 36-45 years reported the highest score of perceived level of competence in performing emergency scale skill (mean rank=126.30), followed by those aged over 45 years (mean rank=119.74) and finally those aged 35 years or less (mean rank=91.82). The difference was statistically significant, p=0.001. None-Saudi physicians had a significant higher score of perceived level of competence in performing emergency skills scale compared to Saudis (mean rank was 120.94 versus 96.67, P=0.009). Physicians who had Saudi Board of Family Medicine (SBFM) reported the highest score of perceived level of competence in performing emergency skills scale (mean score=152.76) whereas those who had Medical bachelors and bachelors of surgery (MBBS) reported the lowest score (mean rank=92.98). The difference was statistically significant, P<0.001. Physicians who reported experience in working in emergency departments had significant higher score of perceived level of competence in performing emergency skills scale (mean score was 111.50 versus 82.59, P=0.002). Physicians who reported more years in PHC practice (>5 years) had significant higher score of perceived level of competence in performing emergency skills scale than those with lower years in PHC practice (<one year) (mean score was 121.90 versus 85.35, P=0.001). Physician`s gender and attending courses of BLS, ATLS and ACLS were not significantly associated with the score of perceived level of competence in performing emergency skills scale.

**Table 2:** Factors associated with perceived level of competence in performing emergency scale skill among PHC physicians

Physician factors	Score of level of competence in performing emergency scale skill(12-48)			P-value

	Median	IQR	Mean rank	
**Age in years:**			
25-35	31	26-73	91.82	
36-45	37	32-42	126.30	
>45	37	27-42	119.74	0.001**
**Gender:**			
Male	33.5	28-38	104.25	
Female	33	26-39	102.98	0.881*
**Nationality:**			
Saudi	32	27-37	96.67	
Non-Saudi	37	28-42	120.94	0.009*
**Qualification:**			
MBBS	31	26-37	92.98	
ABFM^ϕ^	35	33.5-40.5	131.17	
SBFM^ϕϕ^	39	36-44.5	152.76	
FM Diploma	35.5	32-40.75	129.04	
Others	37	23.5-41	109.68	<0.001**
**BLS:**			
<1 year	34	28-39	107.35	
1-2 years	34	28-39	109.14	
>2 years	29	23-38	81.02	
Not at all	25.5	20.75-39.75	75.0	0.092**
**ACLS:**			
<1 year	1-2 years	>2 years	Not at all	
38	34	33	33	
33.75-41	26.5-37.5	28-40.25	26-38	
136.07	100.36	107.67	98.84	0.155**
**ATLS:**			
<1 year	35	27-38	102.09	
1-2 years	35.5	32-36	113.0	
>2 years	36	29-39	117.58	
Not at all	33	27-38.75	101.81	0.727**
**Experience:**			
Yes	35	28-40	111.50	
No	30	24-37	82.59	0.002*
**Years of working in PHC:**			
<1	31	26.25-35.75	85.35	
1-5	32	26.5-37.50	94.96	
>5	36	29-42.5	121.90	0.001**

* Mann-Whitney test;

** Kruskal-Wallis test;

ϕ Arab Board of Family Medicine;

ϕϕ Saudi Board of Family Medicine.

Table 3 shows us the highest rate of satisfaction with all emergency services provided at PHC centers (facilities, equipment, trained personnel and medications) were reported in dealing with acute asthma (47.1%), followed by renal colic (32%) and hypoglycemia (24.3%), whereas the lowest rates were reported in dealing with cases of acute Gastrointestinal tract (GIT) bleeding (4.4%), myocardial infarction (4.9%), cardiac arrest (7.3%), acute vaginal bleeding (7.8%), angina pectoris (8.3%) and convulsions (8.7%). On the other hand, the highest rate of dissatisfaction with all emergency services provided at PHC centers were reported in dealing with cardiac arrest (51.9%), acute GIT bleeding (40.8%), acute vaginal bleeding (39.3%) and myocardial infarction (38.3%). Dissatisfaction with trained personnel (physicians and nurses), the highest rates reported in dealing with cases of hypoglycemia (26.7%), severe dehydration (25.7%), myocardial infarction (23.8%) and diabetic ketoacidosis (23.3%). Dissatisfaction with availability of medications, the highest rates were reported in cases of hypoglycemia (23.3%), renal colic (19.9%), diabetic ketoacidosis (19.9%) and angina pectoris (18.9%). Concerning the dissatisfaction with facilities and equipment, the highest rates were reported in cases of acute GIT bleeding (20.4%), convulsions (19.4%), anaphylaxis (18.9%) and acute vaginal bleeding (18.4%).

**Table 3:** Satisfaction of the PHC physicians with the emergency services provided at PHC centre

Emergency Conditions	Satisfaction with the emergency services provided at PHC centre				

	1 N (%)	2 N (%)	3 N (%)	4 N (%)	5 N (%)
Acute asthma	97 (47.1)	32 (15.5)	36 (17.5)	16 (7.8)	25 (12.1)
Myocardial infarction	10 (4.9)	49 (23.8)	32 (15.5)	36 (17.5)	79 (38.3)
Angina pectoris	17 (8.3)	47 (22.8)	39 (18.9)	34 (16.5)	69 (33.5)
Cardiac arrest	15 (7.3)	39 (18.9)	21 (10.2)	24 (11.7)	107 (51.9)
Severe dehydration	41 (19.9)	53 (25.7)	33 (16.0)	35 (17.0)	44 (21.4)
Renal colic	66 (32.0)	39 (18.9)	41 (19.9)	32 (15.5)	28 (13.6)
Acute GIT bleeding	9 (4.4)	43 (20.9)	28 (13.6)	42 (20.4)	84 (40.8)
Hypoglycemia	50 (24.3)	55 (26.7)	48 (23.3)	26 (12.6)	27 (13.1)
Diabetic ketoacidosis	22 (10.7)	48 (23.3)	41 (19.9)	35 (17.0)	60 (29.1)
Convulsions	18 (8.7)	42 (20.4)	34 (16.5)	40 (19.4)	72 (35.0)
Anaphylaxis	23 (11.2)	47 (22.8)	35 (17.0)	39 (18.9)	62 (30.1)
Acute vaginal bleeding	16 (7.8)	40 (19.4)	31 (15.0)	38 (18.4)	81 (39.3)

1=I am satisfied with the facilities, equipment, trained health care personnel and medications available to deal with such cases.

2=I am satisfied with the facilities, equipment and medications but we need more training for health care personnel (physicians and nurses) when dealing with such cases.

3=I am satisfied with the facilities, equipment and trained health care personnel but medications are deficient when dealing with such cases.

4=I am satisfied with the medications and trained personnel but facilities and equipment are deficient when dealing with such cases.

5=I am overall not satisfied about the services provided at our PHC centre when dealing with such cases.

Table 4 describes the frequencies and percentages of equipments, medications and supporting facilities available for emergency care in 42 PHCCs. Oxygen cylinders with standard fitting, oxygen mask, cannulas were available in all PHCCs. On the other hand, cervical collars were available in only 5 PHCCs (5.9%), urinary catheters in 15 PHCCs (35.7%) and nasogastric tube in 16 PHCCs (38.1%). Medications and intravenous fluids, Dextrose 5%, 10%, 50%, Ringer lactate, normal saline, Ventolin for neublization, Diazepam, Furosemide and Hyoscine were available in all PHCCs whereas Anti-tetanic serum was not available in any PHCC. Activated charcoal powder was available in only 3 PHCCs (7.14%). Supporting facilities, laboratory was available in 41 PHCCs (97.6%) whereas x-ray and equipped ambulance cars were available in 45.2% and 28.6% of PHCCs, respectively.

**Table 4:** Availability of equipment, medication and supporting facilities needed for emergency care at primary health care centers in Jeddah (N=42)

Equipment needed		Medications and intravenous fluid needed		Supporting facilities	

Items	N (%)	Items	N (%)	Items	N (%)
Side lamp with stand	31(73.8)	Calcium chloride injection	16(38.1)	X-ray	19(45.2)
Dressing drum	35(83.3)	Antihistaminic injection	37(88.1)	Laboratory	41(97.6)
Dressing trays	40(95.2)	Hydrocortisone injection	41(97.6)	Equipped ambulance cars	12(28.6)
Dressing table	38(90.5)	Dextrose 5%,10%,50%	42(100)		
Urinary catheter	15(35.7)	Normal saline	42(100)		
Forceps	41(97.6)	Ringer lactate	42(100)		
Scissors	40(95.2)	Activated charcoal powder	3(7.14)		
Suture materials	35(83.3)	Metoclopramide	41(97.6)		
Needle holder	40(95.2)	Adrenaline injection	41(97.6)		
Suction apparatus	24(57.1)	Ventolin for neublization	42(100)		
Blades	40(95.2)	Anti-tetanic serum	0(0)		
IV stand	40(95.2)	Tetanus toxoid	40(95.2)		
Nasogastric tubes	16(38.1)	Diazepam	38(90.5)		
Cannulas	42(100)	Furosemide	42(100)		
Cervical collars	5(11.9)	Hyoscine	42(100)		
Oxygen mask	42(100)				
Airways equipment	19(45.2)				
Oxygen cylinder with Standard fitting	42(100)				
Ambu bag	27(64.3)				
Nebulizer	40(95.2)				

## 4. Discussion

Several studies have shown that emergency situations are to be expected in an active family practice/primary health care office (Klig, 2007; Yorganci & Yaman, 2008). An emergency is “a sudden incident that necessitates urgent and appropriate management to treat its results and avoid its sequelae” (Mahfouz et al., 2007).

In our study, the prevalence of emergency cases among patients attending PHCCs in Jeddah throughout 2013 was 5.2%. Medical emergency cases in PHC in Norway found to be less than 3% (Zakariassen, Hansen, & Hunskaar, 2009). In Netherlands the emergency cases were only 4.6% for the general practitioner (Charante et al., 2007).

This study quantifies the average number and types of emergencies seen in PHC centers in Jeddah, allowing the medical authorities to grasp the importance of improving preparedness of these centers for emergencies. In our survey, more than 70% of the PHC physicians have seen more than three cases of acute bronchial asthma in the last 12 months; in addition frequent cases of renal colic and hypoglycemia were reported. A study done in rural Australia found that the General Practictionars saw a median of eight emergencies per year, and that 95 percent had seen at least one emergency in the preceding 12 months (Johnston et al, 2001). Other study found that the average family practice office has 3.8 childhood emergencies each year (Mansfield et al., 2001). Another study found that 62 % of family medicine and child care offices saw one or more children who required hospitalization or urgent treatment each week (Ablah et al., 2008).

Two thirds of PHC physicians in our study had only attained Bachelor degree level, where the physicians with higher qualification presented an increased score of perceived level of competence in performing emergency skills. Previous studies in Saudi Arabia showed that the majority of PHC physicians would like to acquire more knowledge and skills related to emergency medicine (Almalki, Fitzgerald, & Clark, 2011).

## 5. Concluision

The present study showed that emergency services at PHC level in Jeddah, Saudi Arabia are functioning reasonably well in some terms. However, the services need to be fine-tuned, and defects revealed by the present study should be taken into consideration hand-in-hand with available resources in order to upgrade the quality of the emergency services provided at PHC centers in Jeddah. Level of training and emergency courses of PHC physicians is suboptimal particularly in ATLS and ACLS courses.

Older, non-Saudi, more qualified, more experienced physicians and those having more years in PHC showed higher perceived level of competence in performing emergency skill.
